# Algorithm to improve the diagnosis of paraneoplastic neurological syndromes associated with SOX1 antibodies

**DOI:** 10.3389/fimmu.2023.1173484

**Published:** 2023-05-03

**Authors:** Cristina Arnaldos-Pérez, Andreu Vilaseca, Laura Naranjo, Lidia Sabater, Josep Dalmau, Raquel Ruiz-García, Francesc Graus

**Affiliations:** ^1^ Immunology Department, Centre Diagnòstic Biomèdic, Hospital Clínic, Barcelona, Spain; ^2^ MS Center of Catalonia (CEMCAT), Neurooncology and Autoimmune Neurology Unit, Neurology Department, Vall d’Hebron University Hospital, Barcelona Autonoma University, Barcelona, Spain; ^3^ Neuroimmunology Program, Institut d’Investigacions Biomèdiques August Pi i Sunyer (IDIBAPS), Barcelona, Spain; ^4^ Institució Catalana de Recerca i Estudis Avançats (ICREA), Barcelona, Spain; ^5^ Department of Neurology, University of Pennsylvania, Philadelphia, PA, United States

**Keywords:** SOX1 antibodies, small-cell lung cancer, paraneoplastic neurological syndromes, cell-based assay (CBA), line blot assay

## Abstract

SOX1 antibodies (SOX1-abs) are associated with paraneoplastic neurological syndromes (PNS) and small cell lung cancer (SCLC). In many clinical laboratories SOX1-abs are determined by commercial line blots without confirmation by cell-based assay (CBA) with HEK293 cells expressing SOX1. However, the diagnostic yield of commercial line blots is low and the accessibility to the CBA, that is not commercially available, limited. Here, we evaluated if the addition of the band intensity data of the line blot and the immunoreactivity in a tissue-based assay (TBA) improve the diagnostic performance of the line blot. We examined serum of 34 consecutive patients with adequate clinical information that tested positive for SOX1-abs in a commercial line blot. Samples were also assessed by TBA and CBA. SOX1-abs were confirmed by CBA in 17 (50%) patients, all (100%) had lung cancer (SCLC in 16) and 15/17 (88%) had a PNS. In the remaining 17 patients the CBA was negative and none had PNS associated with lung cancer. TBA was assessable in 30/34 patients and SOX1-abs reactivity was detected in 15/17 (88%) with positive and in 0/13 (0%) with negative CBA. Only 2 (13%) of the 15 TBA-negative patients were CBA-positive. The frequency of TBA-negative but CBA-positive increased from 10% (1/10) when the band intensity of the line blot was weak to 20% (1/5) in patients with a moderate or strong intensity band. Confirmation by CBA should be mandatory for samples (56% in this series) not assessable (4/34; 12%) or negative in the TBA (15/34; 44%).

## Highlights

In many clinical laboratories SOX1-abs are determined by commercial line blots without confirmation by cell-based assay (CBA). However, the diagnostic yield of SOX1-abs detected by commercial line blots is low and the accessibility to confirmatory CBA with HEK293 cells expressing SOX1 is limited.

For sera which are SOX1-abs positive in the line blot and tissue-based assay (TBA) (44%) confirmation by CBA can be spared. Only 13% of samples that are negative in the TBA are positive in the CBA even if the intensity of the band is low-positive.

## Introduction

SOX1 antibodies (SOX1-abs) are serological markers of paraneoplastic neurological syndromes (PNS) associated with small cell lung cancer (SCLC) (1). These antibodies have been included in the group of “high-risk for cancer” antibodies that are important for the definite diagnosis of PNS according to the recently updated diagnostic criteria (2). SOX1 shares a high homology with SOX2 and SOX3, which belong to the group B of the Sry-like high mobility group box family of proteins. SOX1 is expressed in the developing nervous system, the Bergmann glia of the adult cerebellum, and SCLC (3, 4).

In many diagnostic laboratories onconeural antibodies, including SOX1, are determined by commercial line blots. However, the diagnostic yield of this approach is low unless the result is confirmed by another test that usually is a rodent brain tissue based assay (TBA) (5, 6). In the case of SOX1-abs TBA has a lower sensitivity compared to that seen for other onconeural antibodies. Indeed, up to 17% of samples with SOX1-abs confirmed by CBA of HEK cells expressing SOX1 (gold standard test) are negative by TBA (7). However, SOX1 CBA is not commercially available and therefore its accessibility is limited; samples have to be sent to reference centers which overall increase costs and delays in the final diagnosis.

To circumvent these limitations, we evaluated if the intensity of the band of the line blot combined with the presence or absence of reactivity in the TBA improves the diagnostic performance for SOX1-abs without the need of CBA.

## Methods

### Patients and samples

We retrospectively reviewed the clinical data, TBA, and CBA results of 34 consecutive patients with available clinical information whose serum tested positive for SOX1-abs by a commercial line blot assay (Euroimmun, Lübeck, Germany) between January 2018 and December 2022. Serum samples that were positive for additional relevant antibodies were not included. All patients had cancer screening with whole body computed tomography or positron emission tomography scan. Patients were considered as definite PNS according to the 2021 criteria adapted for SOX1-abs based on: 1) presence of a “high risk” or “intermediate risk” neurological syndrome, 2) detection of SOX1-abs confirmed by CBA, and 3) histological confirmation of lung cancer (which is the cancer that associates with SOX1-abs) (2). Patients with neurological syndromes and an alternative diagnosis were not considered PNS regardless of the presence of lung cancer and SOX1-abs.

### Line blot

Serum samples were tested by the commercial line blot EUROLINE Paraneoplastic Neurological Syndromes 12 Ag (DL 1111-1601-7 G; Euroimmun, Lübeck, Germany) following the manufacturers’ instructions at serum dilution 1:101. Test strips were scanned and evaluated for band intensity using the EUROLineScan software (Euroimmun Lübeck, Germany). Following the manufacturer’s recommendations, band intensity values ≤10 were considered negative, values between 11 and 25 were low positive, between 26 and 50 positive, and >50 strong positive.

### Cell based assay

Transfected HEK293 cells with the GFP-tagged SOX1(Origene, RG218236), SOX2 (Origene, RG200757) or SOX3 (Origene, RG210859) clones were fixed with 4% paraformaldehyde for 10 min, permeabilized with 0.3% Triton X-100 for 5 min, and subsequently incubated with patients’ sera, diluted 1:40 in phosphate buffered saline (PBS) with 1% bovine serum albumin overnight at 4°C, and a fluorescent secondary antibody diluted 1:2000 (goat anti-human Alexa Fluor 594 [Jackson ImmunoResearch, PA, USA]) for 1 h at room temperature. Slides were then mounted with Prolong Gold antifade reagent (Invitrogen, Carlsbad, CA, USA) and the reactivity visualized with a Zeiss Axio Imager M2 microscope. Results were scored as positive or negative by two investigators blind to the results of the line blot or clinical diagnosis.

### Tissue-based assay

Adult male Wistar rats were anesthetized and perfused with 0.9% saline solution followed by 2% paraformaldehyde. The cerebellum was further fixed with 2% paraformaldehyde for 4 h, cryoprotected with 20% sucrose in PBS overnight, embedded in optimal cutting temperature (OCT) compound and frozen in methylpropane chilled in liquid nitrogen. Frozen section were air-dried for 30 min and sequentially incubated with 10% normal goat serum for 20 min, patient’s serum (diluted 1:500) for 3 h at 37°C, biotinylated goat anti-human IgG for 30 min, and the avidin–biotin immunoperoxidase complex (Vector Labs, Burlingame, CA, USA) for 30 min. The reaction was developed with 0.05% diaminobenzidine (Sigma, St. Louis, MO, USA) with 0.01% hydrogen peroxide in PBS with 0.5% Triton X-100.

## Results

SOX1 CBA confirmed the presence of SOX1-abs in 17/34 (50%) serum samples that were positive in the line blot. Fifteen of the 17 SOX1 CBA-positive samples were also positive in the CBA of SOX2 and SOX3 whereas none of the 17 samples negative in the SOX1 CBA were positive in SOX2 and SOX3 CBAs. All 17 (100%) patients with SOX1-abs detected by CBA had lung cancer, that was SCLC in 16 (94%), and a definite diagnosis of PNS was established in 15 (88%). The most common PNS was Lambert-Eaton myasthenic syndrome (LEMS) in 6 patients, followed by paraneoplastic neuropathy in 5, encephalitis in 2, and cerebellar degeneration in 2. In contrast, none of the 17 patients with a negative CBA had lung cancer or a PNS (**Table 1**). These patients had isolated symptoms of unclear cause or alternative diagnoses. Four patients had neuropathies, one of them was associated with Sjögren syndrome and another with Waldenström disease. One patient presented neurotoxicity during the treatment with immune checkpoint inhibitors for his prostate cancer (**Table 1**).

**Table 1:** Clinical and laboratory findings in 34 patients with SOX1-abs identified by the commercial line blot.

Patient	Line blot	TBA	CBA SOX1	Lung cancer^&^	Diagnosis
1	56	negative	negative	no	paresthesias
2	47	negative	negative	no	cavernoma
3	43	ANA	negative	no	A-V malformation
4	42	negative	negative	no	fasciculacions
5	38	negative	negative	no	neuropathy
6	32	ANA	negative	no	seizures
7	30	ANA	negative	no	neuropathy
8	24	ANA	negative	no	gait disorder
9	23	negative	negative	no	probable LBD
10	23	negative	negative	no	probable SCA
11	21	negative	negative	no	ICI-related^¶^
12	16	negative	negative	no	seizures
13	16	negative	negative	no	encephalopathy
14	13	negative	negative	no	functional
15	11	negative	negative	no	neuropathy
16	11	negative	negative	no	neuropathy
17	11	negative	negative	no	paquimeningitis
18	62	AGNA	positive	yes	encephalopathy*
19	125	AGNA	positive	yes	LEMS
20	123	AGNA	positive	yes	neuropathy
21	112	AGNA	positive	yes	neuropathy
22	96	AGNA	positive	yes	neuropathy
23	75	AGNA	positive	yes	LEMS
24	68	negative	positive	yes	PCD
25	49	AGNA	positive	yes	LEMS
26	45	AGNA	positive	yes	LEMS
27	44	AGNA	positive	yes	LEMS
28	41	AGNA	positive	yes	neuropathy
29	35	AGNA	positive	yes	encephalitis
30	32	AGNA	positive	yes	brain metastasis*
31	27	AGNA	positive	yes	limbic encephalitis
32	17	negative	positive	yes	LEMS
33	16	AGNA	positive	yes	PCD
34	11	AGNA	positive	yes	neuropathy

AGNA, anti-glial nuclear antibody; ANA, non-neural specific anti-nuclear antibody; ICI, immune checkpoint inhibitor; LBD, Lewy body disease; LEMS, Lambert-Eaton myasthenic syndrome; PCD, paraneoplastic cerebellar degeneration. SCA, spinocerebellar ataxia.

*Non-PNS, ¶ Patient with prostate cancer, & SCLC: 16 patients, neuroendocrine large cell carcinoma: 1.

All 34 samples were also examined by TBA. Four (12%) were not assessable due to the presence of concurrent nuclear antibodies that prevented the evaluation. Immunoreactivity compatible with SOX1-abs (previously defined as AGNA [anti-glial nuclear antibody]) was observed in 15 (44%) samples, all also positive by CBA. The remaining 15 (44%) samples were TBA negative, 13 of them also negative by CBA; the 2 (13%) cases positive by CBA but negative by TBA were from patients with PNS and lung cancer, supporting our previous observation that a small subset of SOX1-abs positive samples are not detected by TBA (1, 7).

**Figure 1:**
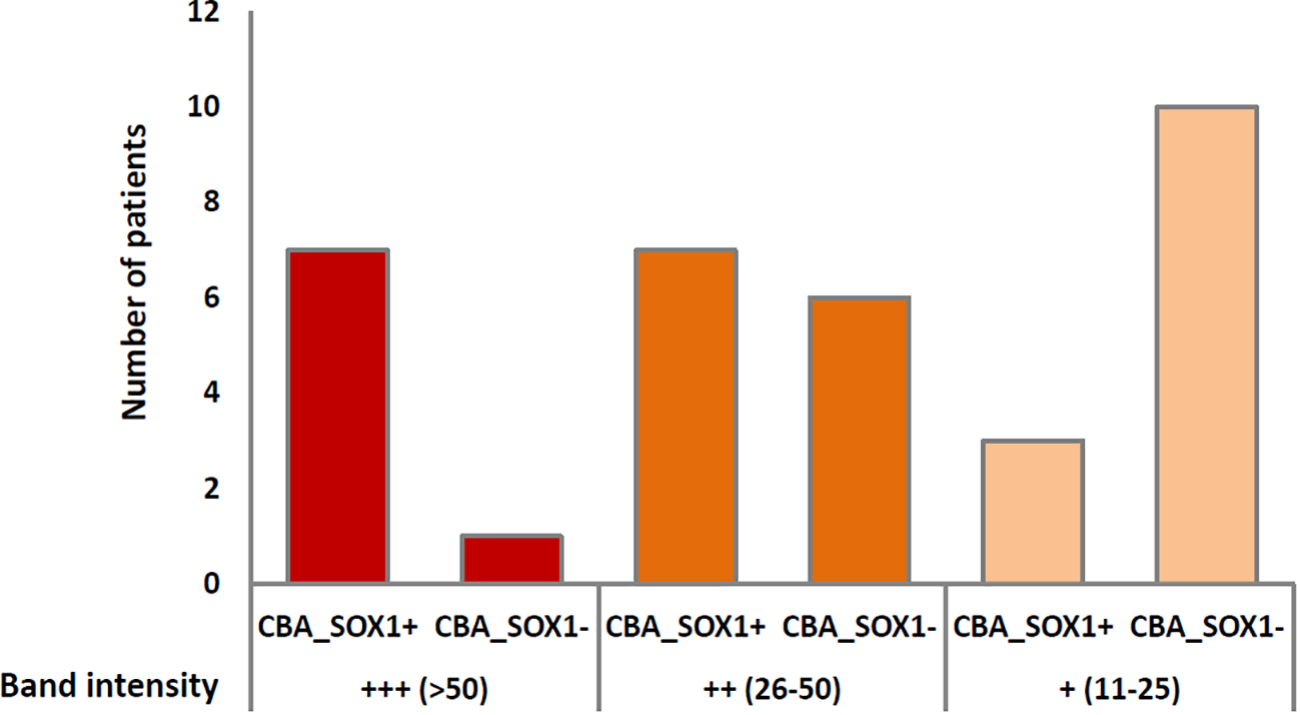
Frequency of CBA-positive samples according to the intensity of the band in the line blot. Numbers in parenthesis refer to the band intensity values provided by the EUROLineScan software.

Sera with a strong positive band of reactivity (>50 EUROLineScan value) were more likely to be CBA positive (7/8; 87%) than those that were positive (26-50 value), 7/13 (54%), or low-positive (11-25 value), 3/13 (23%) (low-positive vs strong positive and positive p= 0.03), (**Figure 1**). We next determined whether the presence or absence of TBA reactivity together with the intensity of the SOX1 band in the line blot were able to predict the CBA results. All 15 patients’ samples positive by TBA and line blot (regardless of the intensity of the band) were also positive by CBA, whereas for the 15 TBA-negative samples, the CBA positivity was not significatively higher in samples with stronger band intensity in the line blot 1/5 (20%) than in those low positive 1/10 (10%) (p >0.05).

## Discussion

**Figure 2:**
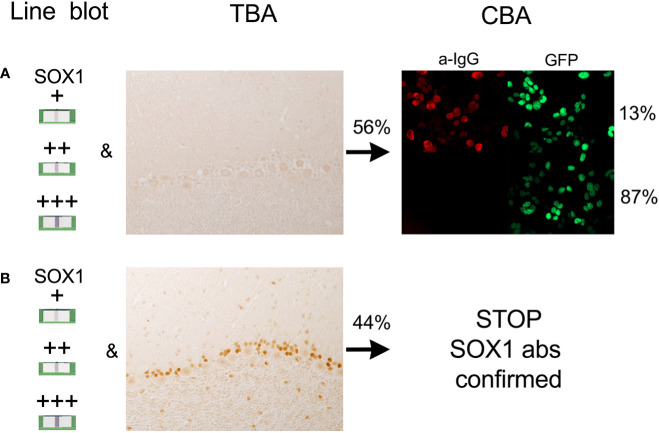
Relation between the findings in the line blot, tissue-based assay (TBA), and cell based assay (CBA). **(A)** Samples positive in the line blot but TBA negative must be tested by CBA as 13% will be positive. **(B)** Samples positive in the line blot and TBA are always CBA positive so CBA could be spared in these samples (STOP). Percentages obtained from the data of 34 patients (see text).

**Figure 3:**
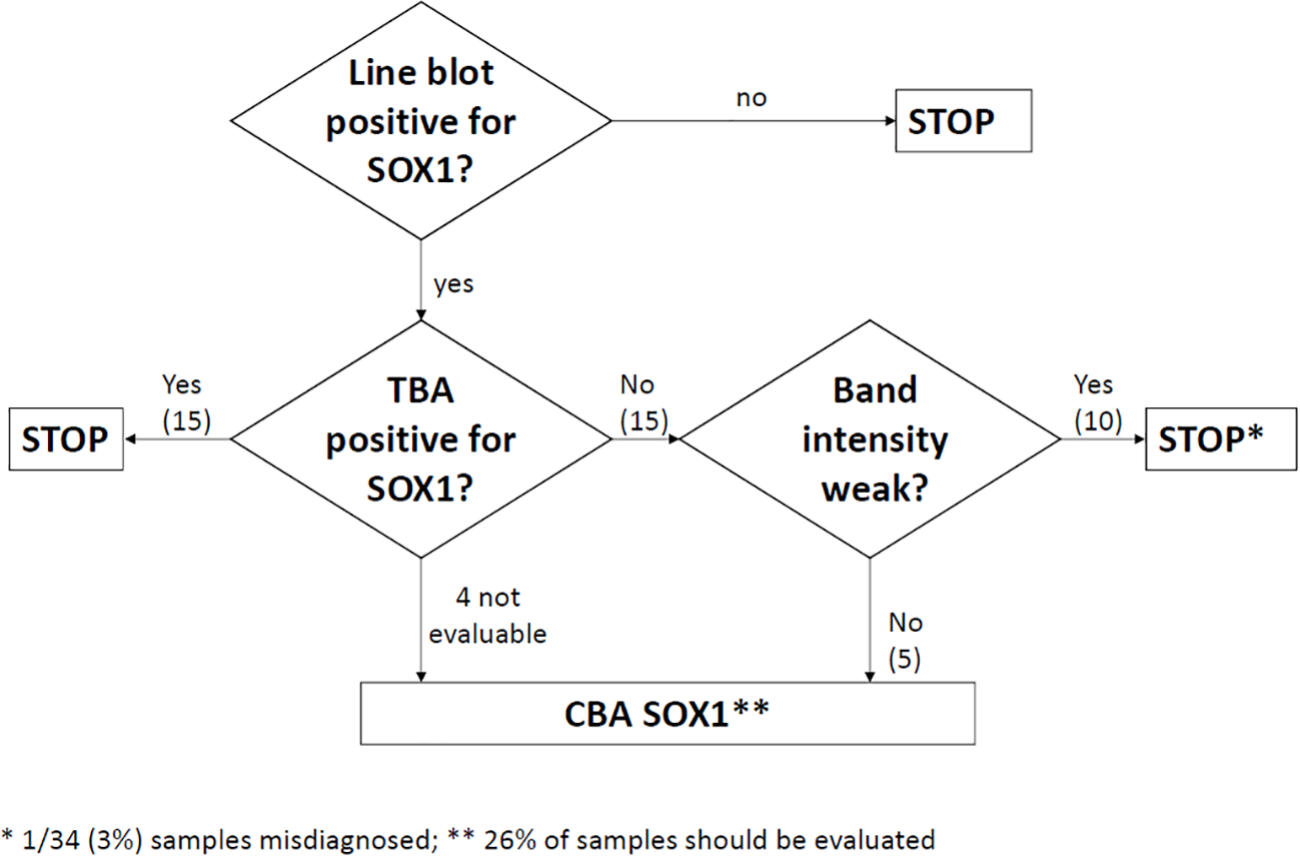
Algorithm considering the possibility to skip CBA testing in TBA negative samples with low positive intensity band in the line blot. The number of samples that should be tested by CBA is reduced to 26% but at the risk to report a false negative result in 3% of samples. Numbers in parentheses obtained from the 34 patients of the study (see text).

The main finding of this study is that all samples with SOX1-abs determined with the commercial line blot and confirmed by TBA were also positive by CBA and associated with lung cancer (100%) and definite diagnosis of PNS (88%). These double-positive samples can be confidently reported as positive for SOX1-abs without need of further studies in reference centers. However, for cases that are line blot positive but are negative or not assessable by TBA, CBA is required. This approach would reduce by about half the number of samples (56% in this series) that need CBA testing (**Figure 2**). An alternative approach is to assess by CBA only the TBA negative samples with positive or strong positive line blot, but not the low-positive line blot cases given that in this group the frequency of a positive CBA is only 10%. Using this alternative approach the number of cases that need CBA is reduced to 26% but at the expense to miss a few CBA positive cases (3% or 1/34 in this series) (**Figure 3**).

The current findings also confirmed our earlier observation that SOX1-abs detected by CBA tightly associate with the presence of PNS and SCLC (1, 7, 8). A recent study suggested that SOX1-abs should be confirmed by 3 assays (line blot, TBA, and CBA) or at least TBA plus either line blot or CBA to support the diagnosis of PNS (9). However, by following the latter suggestion (TBA plus line blot or CBA) 5 of 23 patients (22%) with genuine SOX1-abs and PNS were missed, an observation that is in agreement with our results which show that 2/17 (12%) TBA negative cases had PNS (**Table 1**). In this setting, our findings indicate that a positive result in the line blot must be confirmed by CBA, which is the gold standard assay for SOX1-abs. The discordant results between the two series is probably explained by the lower specificity of the CBA in the study by Vabanesi et al. (9), where a positive CBA was found in 15/18 (83%) patients without PNS whereas in our series all SOX1-abs confirmed by CBA had lung cancer and 88% a definite PNS.

An important observation of our study is that 50% of patients positive for SOX1-abs by line blot were negative by CBA and, unlike those that were CBA positive, they had a wide array of neurological diagnoses without lung cancer association. This finding cast doubts of the true significance of SOX1 antibodies detected in previous studies that reported SOX1 antibodies in non-PNS, mainly neuropathies, as the detection of SOX1 antibodies was done by a single technique (immunoblot or ELISA) and the result was not confirmed by CBA (10–16).

The value of onconeural antibodies positive only in the line blot for the diagnosis of PNS is low unless the result is confirmed by TBA (5). In a previous study we reviewed the clinical diagnosis of 80 patients whose serum was positive for onconeural antibodies by a commercial line blot; in 42 (52%) the antibodies were confirmed by TBA, and 38 (90%) of them had a PNS but only 3 (8%) of the 38 (48%) patients that were negative by TBA had a PNS (6). In the particular case of SOX1-abs, the sensitivity of TBA is lower than that observed for other onconeural antibodies as up to 17% of samples with SOX1-abs by CBA are TBA negative (7). For this reason positive samples by line blot that are TBA negative must be examined with CBA. To circumvent the use of CBA (not commercially available) we examined if the band intensity of the line blot could help to select those cases that despite being TBA negative require CBA. Although we observed a correlation between the line intensity and the CBA result, we were unable to establish an intensity threshold below which all samples are CBA negative; for example, 1 of 10 (10%) samples with low band intensity tested positive by CBA.

The reason why the line blot has a 50% of false positive results for SOX1-abs is unknown and beyond the scope of this study. It could be argued that it is related to the different sensitivity of the assays as the mean band intensity in the line blot of CBA-positive samples was higher than that of CBA-negative. However, there were positive samples by CBA that were low-positive in the line blot and, more important, we previously showed that up to 24% CBA-positive samples from patients with PNS may be negative by the commercial line blot (7). Clinical information of cases SOX1-ab positive by line blot but negative by CBA strongly suggest that are false positive cases, or at least that the antibodies found only by line blot have very limited clinical utility. In our study, none of these cases had PNS associated with lung cancer. To further support this hypothesis, SOX1-abs detected only by the line blot were also SOX2 and SOX3 negative by CBA whereas all CBA-positive SOX1-abs also reacted with SOX2 or SOX3 as previously described (17).

Our study has limitations which require comments. First, our recommendation about which samples can be spared for CBA testing is based on a low number of samples. However, to decrease a potential bias toward PNS and lung cancer we excluded all samples with multiple onconeural antibodies and also those with inadequate clinical information. The second limitation is that we used an in-house TBA. Commercial tissue sections for the detection of onconeural antibodies have not been compared with in-house TBA, thus, it is unclear if the sensitivity and specificity of both TBAs are similar.

Despite these limitations our study demonstrates an important problem in the commercial line blot for SOX1-abs: 50% of results are false positive or misleading. Any positive line blot results must be confirmed by TBA, and those that are TBA-negative by CBA. Therefore, a positive result for SOX1-abs based only in the commercial line blot does not necessarily indicate that the patient has an underlying cancer or should initiate immunotherapy.

## Data availability statement

The raw data supporting the conclusions of this article will be made available by the authors, without undue reservation.

## Ethics statement

The studies involving human participants were reviewed and approved by the ethics committee of Hospital Clínic of Barcelona. Patients’ samples were coded and clinical information was anonymized prior to analysis. Written informed consent for participation was not required for this study in accordance with the national legislation and the institutional requirements. The animal study was reviewed and approved in accordance with European (2010/63/UE) and Spanish (RD 53/2013) regulations by the ethics committee of Hospital Clínic of Barcelona.

## Author contributions

RR-G, JD and FG designed the study and wrote the manuscript. RR-G, CA-P, LN, and LS performed laboratory work and/or data analysis. AV and FG collected clinical information from the patients. All authors contributed to the article and approved the submitted version.
